# Electronic safety-netting tool features considered important by UK general practice staff: an interview and Delphi consensus study

**DOI:** 10.3399/BJGPO.2022.0163

**Published:** 2023-08-23

**Authors:** Claire Friedemann Smith, Sue Duncombe, Susannah Fleming, Yasemin Hirst, Georgia Bell Black, Clare Bankhead, Brian D Nicholson

**Affiliations:** 1 Nuffield Department of Primary Care Sciences, University of Oxford, Oxford, UK; 2 PPI representative; 3 Institute of Epidemiology & Health, University College London, Lancaster Medical School, Lancaster University, Lancaster, UK; 4 Wolfson Institute of population Health, Queen Mary’s University, London, UK

**Keywords:** cancer, patient safety, information technology, primary health care

## Abstract

**Background:**

The potential of the electronic health record to support safety netting has been recognised and a number of electronic safety-netting (E-SN) tools developed.

**Aim:**

To establish the most important features of E-SN tools.

**Design & setting:**

User-experience interviews followed by a Delphi study in a primary care setting in the UK.

**Method:**

The user-experience interviews were carried out remotely with primary care staff who had trialled the EMIS E-SN toolkit for suspected cancer. An electronic modified Delphi approach was used, with primary care staff involved in safety netting in any capacity, to measure consensus on tool features.

**Results:**

Thirteen user-experience interviews were carried out and features of E-SN tools seen as important formed the majority of the features included in the Delphi study. Three rounds of Delphi survey were administered. Sixteen responders (64%) completed all three rounds, and 28 out of 44 (64%) features reached consensus. Primary care staff preferred tools that were general in scope.

**Conclusion:**

Primary care staff indicated that tools that were not specific to cancer or any other disease, and had features that promoted their flexible, efficient, and integrated use, were important. However, when the important features were discussed with the patient and public involvement (PPI) group, they expressed disappointment that features they believed would make E-SN tools robust and provide a safety net that is difficult to fall through did not reach consensus. The successful adoption of E-SN tools will rely on an evidence base of their effectiveness. Efforts should be made to assess the impact of these tools on patient outcomes.

## How this fits in

Electronic tools to support safety netting are being developed and implemented in primary care but are rarely evaluated. This study sought to assess the user experience of a new EMIS E-SN toolkit. It aimed to prioritise existing and additional features of this resource and future E-SN tools through interviews and a Delphi study. It also sought to gather feedback from a PPI group. It was found that primary care staff prioritised E-SN tool features that support the efficient and flexible use of a tool, support decision-making and communication, and would allow for audits to be carried out. While the PPI group did not disagree with these priorities, they stressed that two features considered unimportant by clinician participants (using tools consistently, for every patient, and a notification alerting the GP to repeat consultations in a short time period) would serve to underscore the importance of safety netting and create a robust safety net.

## Introduction

Safety netting is an important diagnostic strategy in primary care to monitor patients with symptoms that could indicate cancer or another serious illness until they are explained or resolved.^1^ Originally conceptualised as a communication skill, safety netting was outlined by Neighbour as a GP asking themselves the following three questions before the close of every consultation: *'If I’m right, what do I expect to happen? How will I know if I am wrong? What would I do then?'* ^2^ Over the past three and a half decades, safety netting has evolved from an exercise for GPs when forming their initial diagnosis and considering the consequences if it is correct or not, to include multiple aspects of diagnostic management, particularly where there is uncertainty. These aspects include clinician–patient communication (what to expect), information provision (what to look out for and where to seek further help), record-keeping, and follow-up.^1,3^ If done well, safety netting can facilitate the safe self-care of patients with self-limiting illness, the earlier identification of serious disease, the avoidance of emergency presentations, and the improvement of patient outcomes.

As cancer is a disease that may manifest slowly and shares many symptoms with benign conditions, safety netting when cancer is a possibility has been a focus for both research and guidance.^4–11^ Despite the widespread acceptance of the central role that safety netting plays in avoiding delays to cancer diagnosis, research has also reported that it is inconsistently used and poorly recorded,^12^ and while the electronic health record (EHR) could support safety netting, its use has not been maximised. Tools to move safety netting online have been created, either directly embedding safety-netting codes or functions into the EHR or facilitating safety netting through EHR integrations with external digital tools such as text messaging^8^ and email.^3^ The purpose of these tools includes helping with GPs’ record-keeping, providing patient information, reminders about follow-up, and improved patient safety by automating, standardising, and centralising safety-netting actions where possible.^3^

E-SN tools have been adopted without an evidence base, however, and there have been few evaluations of their impact on patient safety.^3^ Where E-SN tools have been evaluated, evaluations have stopped short of quantifying any impact they have on outcomes owing to insufficient data,^13^ and have reported conflicting results (Frontier Economics report: *C the Signs evaluation: report for RM Partners* [private report received on request, 2021]).^14^ This qualitative interview and Delphi study is part of the CAncer Safety NETting 2 (CASNET2) study, which aims to fill this gap by evaluating the EMIS E-SN toolkit.^15,16^ E-SN tools have the potential to facilitate the standardisation of safety netting in primary care but these tools must address the needs of the primary care staff who use them, and have a demonstrable, positive impact on patient care. The quantitative evaluation is described in detail separately.^15^

This article presents an evaluation of the E-SN toolkit in the CASNET2 study, including an interview study of user experiences, and a Delphi study to elicit preferences for E-SN system functionality, so that the toolkit can continue to be improved and refined. The objectives of these two studies were, respectively, to explore what determines a GP’s use of the E-SN toolkit, including the barriers and facilitators to the use of E-SN systems, and to identify and prioritise additional desirable features for the CASNET2 E-SN toolkit and E-SN tools generally, as well as features for enhancement.

## Method

This two-part evaluation is centred on primary care clinicians’ views of the CASNET2 tool and E-SN tools more broadly. Patients were not included as participants in the user-experience interviews because this is a clinician-facing tool and a large proportion of its functions are administrative, so patients are unlikely to know that it is being used in their care. The evaluation was supported by PPI involvement throughout.

### User-experience interviews

The procedure for recruiting GP practices to the CASNET2 study, their randomisation, and training on the toolkit is described in detail elsewhere.^15^ Staff from all practices that had trialled the E-SN toolkit, as part of the CASNET2 study, were invited to participate in a user-experience interview. Interviews were partly informed by normalisation process theory (NPT), which aims to identify factors that facilitate or block the adoption of an intervention into usual (or normal) practice.^17^ The four NPT domains were used to shape the questions in the semi-structured interview guide, which allowed for guided questions to be asked on what work was done to implement the toolkit while also allowing the participants to express their preferences and thoughts towards it, for example:

- coherence: how teams were made aware of the toolkit’s uses, and whether the training provided was effective;
- cognitive participation: whether information sharing and uptake was consistent, and what was done to encourage uptake;
- collective action: what using the toolkit was like, if there was anything about it that was a barrier to or facilitator of its use, and how burdensome administrative teams found reporting with the toolkit;
- reflexive monitoring: whether the toolkit had an impact on practice processes, and whether an effect of using the toolkit was seen.

All staff who volunteered were interviewed. The interviews were conducted online using Microsoft Teams by the lead author (CFS) from October–December 2021 and lasted 24–55 minutes. Interviews were conducted online to facilitate the interviewing of staff from across England and to reduce the burden on practices managing the after-effects of the acute period of the COVID-19 pandemic.

The interviews began with a discussion about the interviewee’s general practice and role. The discussion then turned to how staff were trained on the toolkit, how it was received, and the features that the interviewees liked, disliked, and felt were missing. These broad themes were also pre-specified for the data analysis (Supplementary Figure S1). The interviews were transcribed verbatim and analysed using a framework analysis by CFS. Framework analysis allows for both inductive and deductive thematic analysis of qualitative data, and was selected for the set of pre-defined research questions to be answered and also to allow unanticipated themes to be generated.^18^ Weekly meetings were held at which CFS updated the research team on progress and discussed the developing findings. Following the interviews, the features brought up, along with others mentioned in the literature, were presented to the study team. This generated the statements included in the first round of the Delphi survey.

### Delphi survey study

A three-round modified Delphi method was used for this study. The Delphi method aims to reach a consensus of expert opinion on a topic through iterative rounds of questioning and feedback, and commonly involves 10–50 expert panel members.^19^

### Sample

Any individual working in primary care who was involved in the safety netting of patients in any capacity was eligible to take part. The invitation to participate in the Delphi survey was disseminated through a number of channels including the following: invitations to practices participating in the CASNET2 study; the Society for Academic Primary Care (SAPC) newsletter and Twitter account; Macmillan Cancer Support; North Central London Cancer Alliance; Cancer Research UK; Royal College of General Practitioners (RCGP) newsletter; the RCGP Research and Surveillance director’s message newsletter; and the Twitter accounts and personal contacts of the study team. All invitations included a link to materials explaining the purpose and process of the study. As the safety-netting tools under discussion are intended for primary care staff to use, no patients were included in the Delphi panel. However, a PPI group meeting was held to discuss the perceived impact of the tool features on patient care (see below).

### First survey round

Three rounds of an electronic survey were used (Supplementary Figures S2–S4), hosted on Online surveys (https://www.onlinesurveys.ac.uk/). The first-round survey was developed based on the user-experience interviews and published literature on electronic tools to follow-up patients post-discharge, tools for clinical decision support, and risk prediction tools.^20–28^ The initial items for the survey were developed by CFS and shared with the study team who amended and added items from their experience as clinicians and researchers. All survey rounds were distributed via email.

Forty items detailing features of E-SN tools were grouped into the following seven themes: information entry; integration with other systems; communicating with patients; warning notifications; task-setting and follow-up; responsibility for safety netting; and closing the safety-netting record and auditing. The Delphi panel was asked to rate how important they believed each of these features were to a tool that would facilitate effective safety netting on a 5-point Likert scale where 1 indicated 'not at all important' and 5indicated 'very important'. Additionally, the panel were asked to rank how useful an e-safety-netting tool would be for different types of presentations (for example, disease specific versus general) and for different patient groups (for example, those with an urgent referral versus those who do not meet the referral criteria yet). The opportunity to leave free-text comments was provided.

### Analysis

The proportion of the panel rating each feature as important (Likert scale rating 4 or 5) or unimportant (Likert scale 1 or 2) was calculated. Consensus was defined as at least 75% of the panel rating a feature as 'important' or 'very important' ('consensus important'), or 'unimportant' or 'not at all important' ('consensus unimportant'). If consensus was achieved, it was ranked as 'adequate' (75%–79%), 'strong' (80%–84%), 'very strong' (85%–89%), or 'overwhelming' (90%–100%). Free-text comments were analysed for new features or amendments to existing features. This was repeated for each survey round.

### Second and third survey rounds

In the second and third survey rounds, individuals who had responded to the previous round were emailed the subsequent survey. Features remained in their themes but were grouped as those that had achieved consensus and those that had not. Recipients were given a copy of their previous responses for reference. If features were added or amended, an explanation was given. Where a feature had not achieved consensus, a summary of how the panel had voted and a selection of anonymous comments given in the previous round was presented (where available). The panel were then asked to rate the importance of the feature again. Any features that had not reached consensus after the third round were defined as not having achieved consensus.

### PPI group involvement

Five members of the public were recruited through cancer and health research networks in early 2020 to the CASNET2 PPI group. The first PPI group meeting was scheduled for March 2020 but owing to the COVID-19 pandemic, which delayed the start of the CASNET2 study as a whole, the first meeting was held online in September 2020. During the early stages of the study, the group met online approximately every 6–8 months and this increased as results and next steps have became ready for discussion. Two PPI meetings were held following the completion of the user interviews and Delphi survey studies. At the meeting following the user-experience interviews, the results were fed back to the group and discussed, and the process of a Delphi survey study explained. During this meeting the group were asked to comment on what the interviewees had said and indicate what features they thought were important or less important before the Delphi items were drafted. Following the Delphi survey, a meeting was held at which the features that achieved and did not achieve consensus were discussed in depth. The group shared what they thought the implications could be for patient care if E-SN tools were adopted that were designed in line with the 'important' and 'unimportant' features. A summary of this meeting is presented in the Results section.

## Results

### User-experience interviews

The demographic characteristics of all 13 primary care staff who volunteered and took part in the user-experience interviews are given in Table 1. The majority of the interviewees thought the CASNET2 toolkit had been useful and suggested that they would like to continue using it after the trial ended. The interviewees said they believed the toolkit was useful to standardise safety-netting practice, if used consistently. Although all of the interviewees made suggestions about ways the toolkit could be improved, a small number said that significant changes would need to be made before they would be willing to incorporate it into their practice permanently.

**Table 1.** Characteristics of user-experience interview participants

Participant ID	Sex	Years’experience	Role in practice
P01	F	13	GP
P02	M	2 (with practice)	Research physician
P03	F	7	GP
P04	F	20	GP
P05	M	38	GP
P06	F	15	Administrator
P07	F	15	GP
P08	F	21	Advanced nurse practitioner
P09	M	20	GP
P10	M	19	GP
P11	F	25	GP
P12	F	Not stated	Research nurse
P13	F	15	GP

The suggestions for areas of improvement could be broadly separated into improving the user interface and providing the user with information. Improvements to the user interface included the following: reducing the number of ‘clicks’ needed to complete forms; increasing the extent to which the form auto-populates; and improving its integration while reducing any overlap with other systems. In terms of the information, the interviewees wanted the toolkit to provide visual alerts when dates associated with an instance of safety netting were breached; guidance on appropriate safety-netting timelines (for example, how long to wait for contact from secondary care, or for a symptom to resolve); and the ability to see how, or if, use of the toolkit was benefitting the patient. A summary of the interview themes with illustrative quotes is available in Supplementary Figure S1.

### First-round Delphi survey

The first-round survey was sent to 25 primary care staff who had responded through an expression of interest form. It was circulated in February 2022 and received 20 responses (80%). The characteristics of those responding to the first round are given in Table 2.

**Table 2.** Characteristics of responders to the first round Delphi survey

Characteristic		
**Sex**	Female	8 (40%)
	Male	12 (60%)
**Mean age, years (range)**	47 (31–66)	
**Role within practice**	GP partner	9 (45%)
	Salaried GP	5 (25%)
	Locum GP	3 (15%)
	GP trainee	1 (5%)
	Practice manager	1 (5%)
	Other	1 (5%)
**Holds an academic as well as clinical role**	Yes	10 (50%)
	No	10 (50%)
**Mean years since qualification (range)**	19 (4–43)	
**Safety-netting tools ever used^a^**	E-SN toolkit	6 (30%)
	Ardens	10 (50%)
	Accurx	9 (45%)
	C the Signs	3 (15%)
	None	5 (25%)
**Safety-netting tools currently using^a^**	E-SN toolkit	2 (10%)
	Ardens	7 (35%)
	Accurx	9 (45%)
	C the Signs	1 (5%)
	None	7 (35%)

^a^Responders could select more than one answer. E-SN = electronic safety-netting toolkit

In round 1, 12 of the 40 features (30%) reached consensus. For all but one the consensus was that the features were important, and in one there was consensus that it was unimportant. In light of the free-text comments, two statements were amended for clarity and three additional features were added. A selection of the free-text comments is available in Supplementary Figure S5.

### Results from survey round 2

Eighteen responses were received to the second survey round and an additional 10 features achieved consensus. Following the free-text responses, one statement was amended for clarity and one feature was added.

### Results from survey round 3

Sixteen responses (64% responding to all three rounds) were received for the third and final round and an additional six features reached consensus. By the end of round 3, 28 out of 44 features (64%) had reached consensus. More than half of the statements that did not reach consensus were features that could support informational continuity of care; for example, ‘Creates alert if patient attends repeatedly in a short timeframe’ (number 23) and ‘Pop-up message boxes on opening patient record if follow-up date has passed’ (number 32). Supplementary Figure S6 presents the feature statements and the results of all three survey rounds.

**Figure 1.**
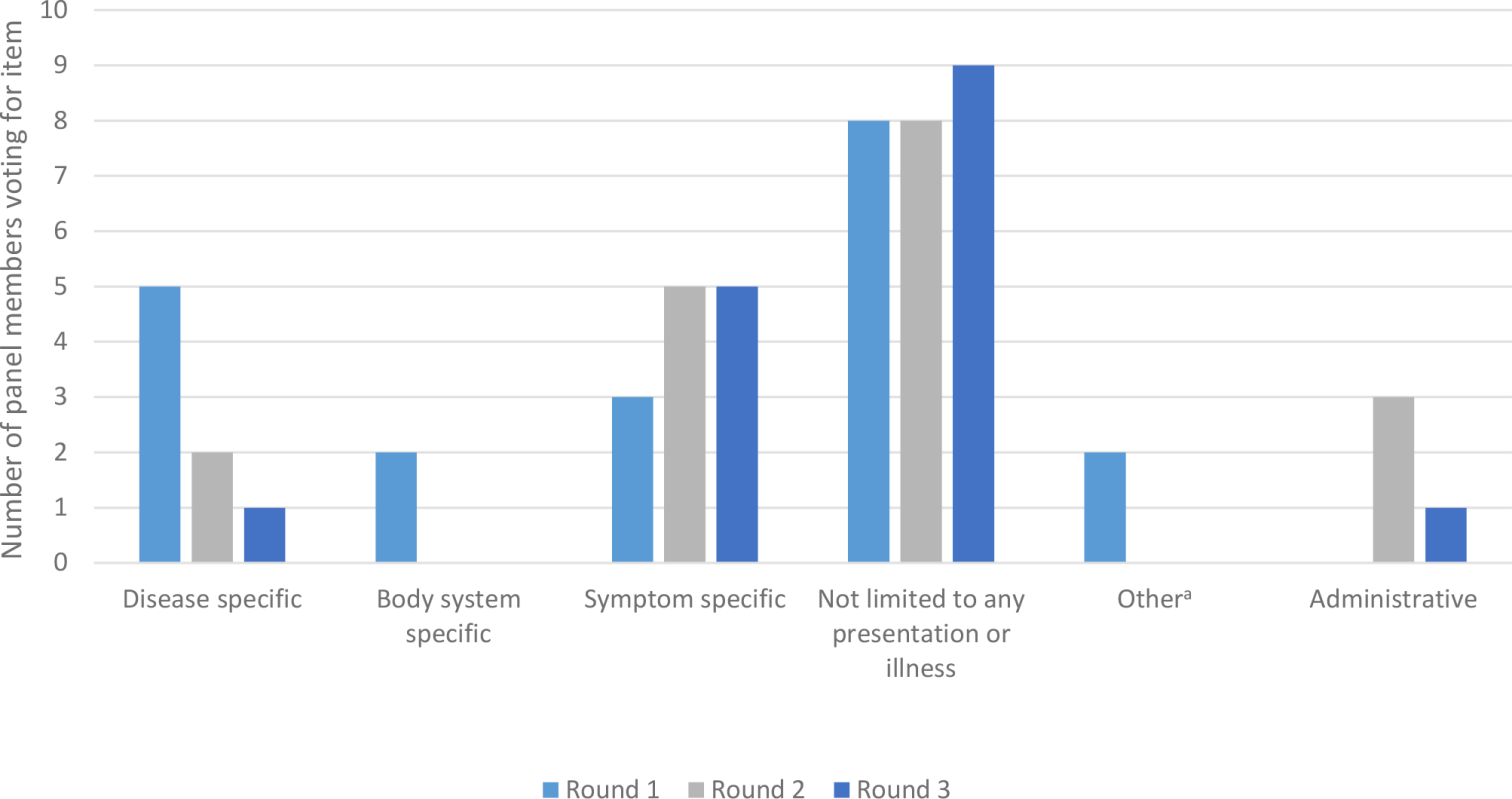
Votes for preferred scope of e-safety-netting tool. ^a^’Other’ allowed participants a chance to suggest other areas where an E-SN tool would be useful. This was selected twice in round 1 by one participant, who said that they would rather not use the tools and another who suggested Administrative. Administrative was added to the subsequent rounds.

**Figure 2.**
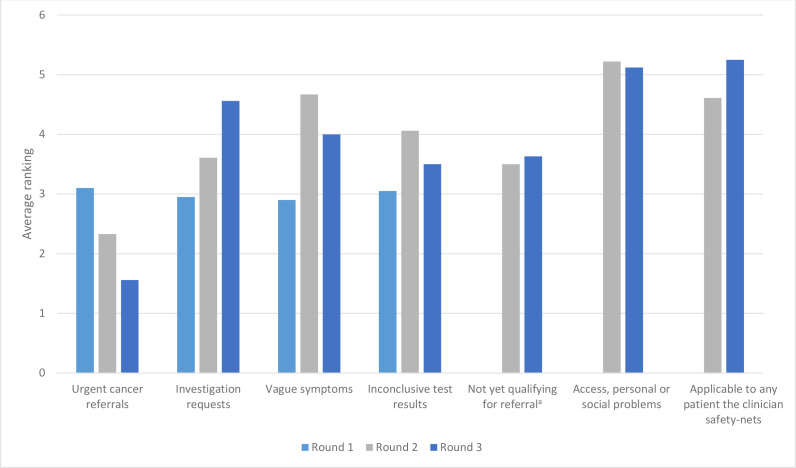
Average ranking of patient groups for whom of e-safety-netting tool would be most useful. In round 1, patient groups were ranked on a scale of 1–4, in rounds 2 and 3 on a scale of 1–7. ^a^These patients were described as, for example, those who had not had their symptoms long enough to meet the referral threshold.

Two additional questions were asked in all three survey rounds regarding the scope of E-SN tools (Figure 1) and the patient groups for which tools were considered most useful (Figure 2). These questions were included to try to find a consensus on the circumstances in which, and patients for whom, E-SN tools were considered useful. The participants responded that tools that were most generally useful; that is, those that could be used with any patient that the clinician chose to safety net, and could facilitate safety netting for a range of patient presentations. Additionally, tools that could facilitate safety netting for patients that have access, personal, or social problems were ranked highly.

### PPI feedback

The outcomes of the Delphi survey were presented to the PPI group to understand what the implications for patients might be if E-SN tools were designed to incorporate the Delphi study priorities. The agreed statements were uncontroversial and accepted, with one exception. The PPI group were disappointed that the statement ‘Use of tool is compulsory for all patients’ (Statement 1, Supplementary Figure S6) was deemed unimportant. The PPI group suggested that making tools optional would erode their use over time and contradicted the message that safety netting was an important part of the consultation.

For the statements that were not agreed, there was tension between clinicians’ desire for E-SN tools not to introduce 'superfluous' information or create additional tasks, and the PPI groups' opinion that these features contributed to a more effective safety net. The PPI group raised concerns that the rationale for not rating some statements as important could not be applied to all patients. For example, Delphi participants reported that the clinician would notice that a patient had attended multiple times within a short period or with the same symptoms when reviewing the patient’s notes rather than requiring a notification, and that repeat consultations are rarely because of serious illness (Supplementary Figure S5). The PPI group, however, said that this assumes the clinician has time to thoroughly review the patient’s notes before the consultation or that the patient is able to reconsult with the same clinician, either of which may not be possible. The PPI group stated that even if repeat attendances for the same symptom are rarely related to serious undiagnosed illness, the rare case is exactly what safety netting is for. Another feature that was not rated important was the provision of evidence-based guidance for the expected duration of non-specific symptoms to guide self-management advice. The PPI group agreed that standardisation could be difficult for some non-specific symptoms, but stressed that where evidence was available an attempt should be made as standardisation reduces variation in advice and reassures patients, echoing previous research.^29^

## Discussion

### Summary

In this study, the Delphi panel agreed on 28 features of E-SN tools that they believed to be either important or unimportant for effective safety netting. The features that reached consensus were generally those that could support the efficient and flexible use of a tool, would support decision-making and communication with patients and colleagues, and would allow for the impact of the E-SN tool to be assessed. Several items relating to task-setting and follow-up did not reach consensus. The panel also preferred safety-netting tools that were not targeted at specific diseases, body systems, or symptoms.

Generally the features that reached consensus quickly were those that had a bearing on the clinicians’ time; for example, use of auto-population. In contrast, those that took two or three rounds of Delphi to reach consensus were generally more relevant to the safety implications of the E-SN tool. Further, the majority of features that did not reach consensus were those that provided information and supported informational continuity of care. This implies that concerns over burden of work and timekeeping may have been more salient to the participants than the mitigation of risk. While this is understandable given the current pressures on primary care, and tools that reduce administrative burden are needed, it is equally important that tools should be effective at improving patient safety.

### Strengths and limitations

The majority of the Delphi features were derived from interviews with primary care staff who were trialling an E-SN tool. As such, the statements were based on their recent experiences of using an E-SN tool, increasing the likelihood that the features included were relevant to current primary care practice. The Delphi panel was balanced in terms of those who held academic positions and those who did not. It is conceivable that academic primary care clinicians could hold different views to those focused solely on clinical work so the even split is a strength. The panel also included clinicians with experience of a range of current E-SN tools, ensuring that the strengths and weaknesses of a range of tools were reflected in the results.

Only 13 primary care staff were recruited to the user-experience interviews despite a concerted effort by the RCGP Research and Surveillance Centre practice liaison officers administering the CASNET2 study. This may be because recruitment occurred in April–November 2021 while UK government restrictions were still in place and the acute phase of the COVID-19 pandemic was coming to an end. It has been well documented that the pandemic placed huge pressures on primary care, which may have prevented staff from taking part in non-essential activities such as the interviews.^30,31^ However, the study had a narrow remit for these interviews, which was to discuss the work done to implement the E-SN toolkit and the experience of using it, with a specific group of participants — primary care staff who had used the toolkit — and so the informational power^32^ may have compensated somewhat for the small sample size.

Although any individual working in primary care involved in safety netting was accepted into the study, the majority of the participants were GPs. It is clear from the user interviews and the comments provided in the Delphi study that aspects of safety netting and follow-up are carried out by non-clinical staff, but their preferences have not been captured in this study. Additionally, professionals from outside of primary care were not included; for example, patient safety experts or clinicians from acute settings. As such the results of the Delphi may reflect and be limited by current primary care safety-netting practice.

Patients’ views also did not directly contribute to the prioritisation of E-SN tool features. The PPI group have contributed throughout the CASNET2 study, helping to set and prioritise outcomes for the quantitative evaluation of the E-SN toolkit, commenting on the findings of the user-experience interviews, and by supporting or opposing the findings of this Delphi study. As such, it is believed that their views have been and will continue to be meaningfully incorporated into the continuing development of the E-SN toolkit. Finally, the panel size was at the small end of what is considered acceptable for Delphi studies. However, small but homogeneous Delphi panels can produce high quality consensus.^19^

### Comparison with existing literature

The adoption of EHR systems, which has happened alongside the expansion of safety netting, should have provided new ways to support safety netting, for example through electronic alerts,^33^ but the literature has shown that safety netting is often kept ‘offline’.^12^ The authors of the present study have found clinicians to be in favour of electronic safety netting in this and in previous work.^30^ However, as in previous research,^27,28,34^ the authors were also told that higher volumes of tasks and alerts leads to clinicians ignoring them, and heard calls to simplify, automate, and 'stop the pop'; that is, limit pop-up notifications. The free-text responses in this study support previous studies that describe disagreement around what safety netting is, how it should be used, and whose responsibility it is to ensure advice is followed.^6,7^ Furthermore, a number of the participants commented that follow-up was a separate issue to safety netting despite previous research that has not made this distinction.^12^

A recently published framework outlined nine principles that denote a high quality E-SN tool.^3^ The features that reached consensus reflected many of the principles outlined in this framework (Table 3), in that E-SN tools should facilitate shared responsibility for safety netting; should integrate well with the EHR and automate data capture; should facilitate quality improvement; and should allow information sharing with the patient. This framework also includes principles such as that the tool should be used with all patients not only those thought to be high risk, and that it should track patient consulting patterns and create alerts. Similar features did not reach consensus in the Delphi study, perhaps highlighting a gap between what might theoretically support safety netting and what, in practice, will become too burdensome within the consultation. Ways to reduce the burden of such features on staff should be investigated.

**Table 3.** The Delphi items^a^ reaching and not reaching consensus that met the nine principles of high-quality safety-netting tools

Nine principles of E-SN tools (reproduced from Black *et al*)^3^	Features reaching consensus that met the principle	Features not reaching consensus that met the principle
All patients registered will be e-safety netted.	Scope and patient groups where a tool was considered most useful.	1. Use of tool is compulsory for all patients (consensus was reached but it was that this item was not important).9.Use of tool compulsory to create a consistent record of when a patient is safety- netted.
All clinicians and primary care staff are responsible for e–safety netting.	38. Centralised record of safety netting seen by all practice staff to share the responsibility.	
Limit burden and cognitive bias by using automatic functions, where possible.	2. Auto population of fields when information is already in the health record.6. Automatic triggering of e-safety-netting tool based on coding.29. Automatically sends message to administration team when follow-up timelines are breached.	35. Automatically creates diary entry for follow-up.
Support diagnostic processes before, during, and after consultations.	12. Links to patient information leaflets.14. Links to the up-to-date referral criteria for 2ww pathways provided in the form.16. Ability to send safety-netting advice and follow-up timeline to patients.17. Editable templates of text messages to send to patients.19.Ability to send texts to patients.30. Reminders for routine follow-up of patients that can be scheduled as diary entries.	23. Creates alert if patient attends repeatedly in a short timeframe.24. Creates alert if patient attends repeatedly for the same symptoms.25. Notifies user of time since last consultation.31. Pop-up message boxes on opening patient record detailing any safety-netting advice that has not been closed.32. Pop-up message boxes on opening patient record notifying if follow-up date has passed.
Monitor, auto-detect, and measure pathway process errors, or deviations and alert the relevant people.	21. Creates an alert on opening patient record if patient has not been investigated or a result has not been returned.22. Alerts take the form of an automatically generated urgent task (for example, to follow-up with the patient).	
Use simple processes that make it easy to access and transfer complex information.	11. Integrates with other systems to avoid duplication of work.13. Test results are automatically incorporated so clinician doesn’t have to look elsewhere.	33. Guidance on the duration vague symptoms should be managed by the patient before they are advised to re-contact the GP.
Spread responsibilities and roles within primary care that have an overall impact on the whole patient pathway.	36. Ability to assign ownership for safety-netting actions.37. Ability to electronically ‘hand-over’ responsibility for the safety netting to a colleague.38. Centralised record of safety netting seen by all practice staff to share the responsibility.	34. Automatically sends message (for example, email) to responsible clinician when follow-up timelines are breached.39. Automatically sends tasks to relevant staff members.
Support senior leadership to optimise safety strategies within a regular quality improvement programme.	41. Ability to audit use of the e-safety-netting tool.	44. Automatically produces reports of breached follow-up timelines.
Allow for patient interaction and feedback.		20. Ability to automatically send test or investigation results to the patient.

^a^A small number of items did not reflect the principles and are not included here.

2ww = 2-week wait referral.

### Implications for research and practice

This research has highlighted features of E-SN tools that clinicians believe to be important or desirable and how the PPI group believe these and the omitted features will impact the provision of safety netting. E-SN tools will continue to evolve and future tools should incorporate these preferences and measure whether user experience and patient safety is improved. Widespread adoption of E-SN tools will be facilitated by, and may be dependent on, strong underpinning evidence, as highlighted by the Delphi participants and in previous research.^28^ Assessing the impact of tools on patient outcomes, such as rates of appropriate reconsultation, the number of consultations before referral for 2-week-wait investigation, and the number of missed tests or missing test results, is an important part of this evidence base. Additionally, gathering patient feedback on the experience of being safety netted, what in the interaction works well, and what needs to be improved is important for the provision of safety-netting advice generally. Standardised and objective frameworks should also be used to identify high quality E-SN tools. Both of these topics are under-researched with, to the authors' knowledge, only one (as yet untested) quality framework,^3^ and one study exploring the impact of E-SN tools on patient outcomes that is yet to report findings.^15^

There was a tension between the preference of GPs to have safety-netting tools that are simple, flexible, and unobtrusive, and the PPI group who wanted safety netting to be standardised and to use more alerts and warning notifications that aim to make the safety net difficult to fall through. As E-SN tools continue to develop, patient preferences should be taken into account and new ways need to be found that can support the universal and robust provision of safety netting that patients want, without placing too great a burden on clinicians. An example of how this may be achieved is through the targeted and intelligent use of technology to ensure tool functions are meaningful. Targeted and intelligent technology could, for example, identify the subtle increases in healthcare contacts that have been shown to occur in the months leading up to a cancer diagnosis.^35–38^ E-SN tools could also monitor consultation patterns and create an alert if the pattern for the individual patient changes over time. This approach may reduce inappropriate alerts for patients who usually attend frequently while also providing alerts if the individual’s presentation patterns change.

The increasing use of the EHR has presented an opportunity to move safety netting from a verbal exchange, perhaps recorded in a note, to an online systems-based approach. Successful E-SN will depend on the development of tools that are intuitive to use, provide a level of informational continuity even if there is no relational continuity, and are supported by evidence of improved patient outcomes. This research has established consensus among primary care clinicians on the features of E-SN that are important. Future research should incorporate these features into E-SN tools and gather evidence of their impact and the benefits to patients.

## Data

The dataset relied on in this article is available from the corresponding author on reasonable request.
